# Persistent Pupillary Dilation and Irreversible Mydriasis in HLA-B27-Associated Uveitis

**DOI:** 10.7759/cureus.89830

**Published:** 2025-08-11

**Authors:** Hamdah M. Alkhaldi, Wael A Alsakran, Moustafa S Magliyah

**Affiliations:** 1 Medical Retina and Uveitis, King Khaled Eye Specialist Hospital, Riyadh, SAU; 2 Vitreoretina and Neuro-ophthalmology, King Khaled Eye Specialist Hospital, Riyadh, SAU

**Keywords:** atonic pupil, irreversible mydriasis, paretic pupil, urrets-zavalia-like syndrome, urrets-zavalia syndrome

## Abstract

Persistent fixed dilated and tonic pupils following an attack of anterior uveitis is an uncommon finding, particularly in patients with a confirmed diagnosis of human leukocyte antigen B27 (HLA-B27)-associated uveitis. The proposed mechanism in this condition is linked to anterior segment ischemia, a phenomenon known as Urrets-Zavalia-like syndrome. We describe a case of a healthy 38-year-old female patient who presented to the neuro-ophthalmology service with persistently dilated pupils in both eyes. She had a past ocular history of recurrent anterior uveitis that was later associated with an HLA-B27 mutation. During the acute attack of anterior uveitis, the patient received topical cyclopentolate 1% drops as part of her management. Later, she presented with worsening photophobia and high ocular pressure, in addition to widely dilated and fixed pupils (atonic pupils) in both eyes. Upon assessment during follow-up visits, the pupils were found to be persistently dilated even after the discontinuation of the mydriatic drops and stabilization of both ocular pressure and inflammation.

## Introduction

Irreversible mydriasis, although most commonly reported following ocular surgeries such as penetrating keratoplasty (Urrets-Zavalia syndrome), can rarely occur in the context of ocular inflammation, particularly anterior uveitis, complicated by elevated intraocular pressure (IOP) [1,2]. The underlying mechanism is thought to involve ischemic injury to the iris sphincter muscle due to an acute rise in IOP, leading to permanent damage and loss of pupillary function [3]. Inflammatory debris can obstruct aqueous outflow, causing pressure spikes that compromise iris perfusion. This ischemic insult is analogous to mechanisms observed postoperatively and results in fixed, dilated pupils that do not react to light or near stimuli. Iris atrophy and transillumination defects may be observed on slit-lamp examination, supporting the diagnosis [1]. Human leukocyte antigen B27 (HLA-B27)-associated acute anterior uveitis (AAU) constitutes the most frequent identifiable cause of non-infectious uveitis, accounting for roughly half of all AAU [4].

Clinically, it is essential to differentiate this ophthalmic cause from neurologic etiologies such as third nerve palsy or midbrain lesions. Unlike neurologic causes, ischemia-related mydriasis typically presents without ptosis, extraocular movement abnormalities, or altered consciousness [5,6]. In such cases, the chronology of anterior uveitis followed by an IOP spike and subsequent mydriasis, in the absence of neurological signs and synechia, strongly supports a local ocular cause.

## Case presentation

**Figure 1:**
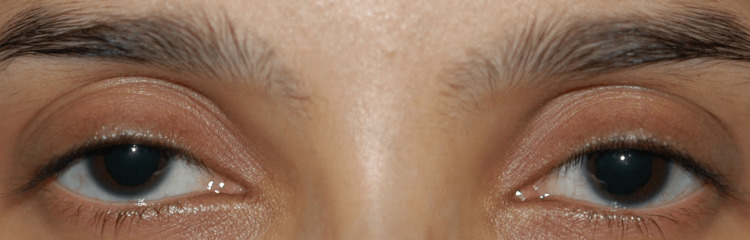
External photograph showing symmetrical dilation of the pupil in both eyes. Written informed consent to include the image in an open-access article was obtained from the patient.

**Figure 2:**
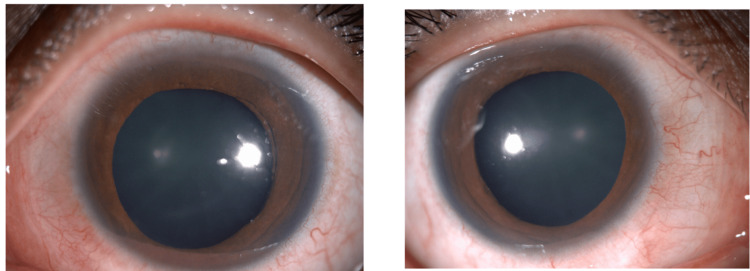
Close-up photographs with diffuse illumination technique highlighting the widely dilated pupils.

A 38-year-old medically free female patient was referred to our institute after complaining of a recent decrease in vision and photophobia bilaterally. On initial examination, the vision in each eye was 0.3 logMAR, and the IOP was 16 mmHg in the right eye and 18 mmHg in the left eye. The anterior segment examination was remarkable for bilateral moderate conjunctival and ciliary body hyperemia, in addition to grade +2 anterior segment inflammation. The pupil assessment was unremarkable on initial assessment, with symmetrical reactivity to both light and near targets. The other components of optic nerve function testing, including color vision and confrontational visual fields, were normal for both eyes. The fundus assessment appeared normal with a cup/disc ratio of 0.3 bilaterally, with no signs of posterior segment inflammation. A full uveitis work-up was within normal except for high titers of HLA-B27, and she was treated with topical 1% prednisolone acetate as well as topical 1% cyclopentolate drops (Figures 1-2).

One week after the presentation, the patient reported moderate ocular pain and light sensitivity in both eyes. Her examination was remarkable for high IOP (29 mmHg in the right eye and 32 mmHg in the left eye), in addition to medically dilated pupils (6 mm pupil size). However, the inflammation was noted to improve compared to presentation (grade +1, bilaterally). Angle assessment indicated a crowded angle in all quadrants due to mydriasis, with no peripheral anterior synechiae. Anti-glaucoma drops were added to manage the elevated IOP, and mydriatic drops were discontinued to reduce the pupil size, which could improve her light sensitivity. Two weeks after the first follow-up, the IOP was unremarkable, and her assessment indicated complete resolution of inflammation. However, the pupils were persistently dilated bilaterally with no reaction to light or near, even after two weeks of halting the cyclopentolate drops. The patient was referred to neuro-ophthalmology to address the persistent and fixed dilated pupils.

**Figure 3:**
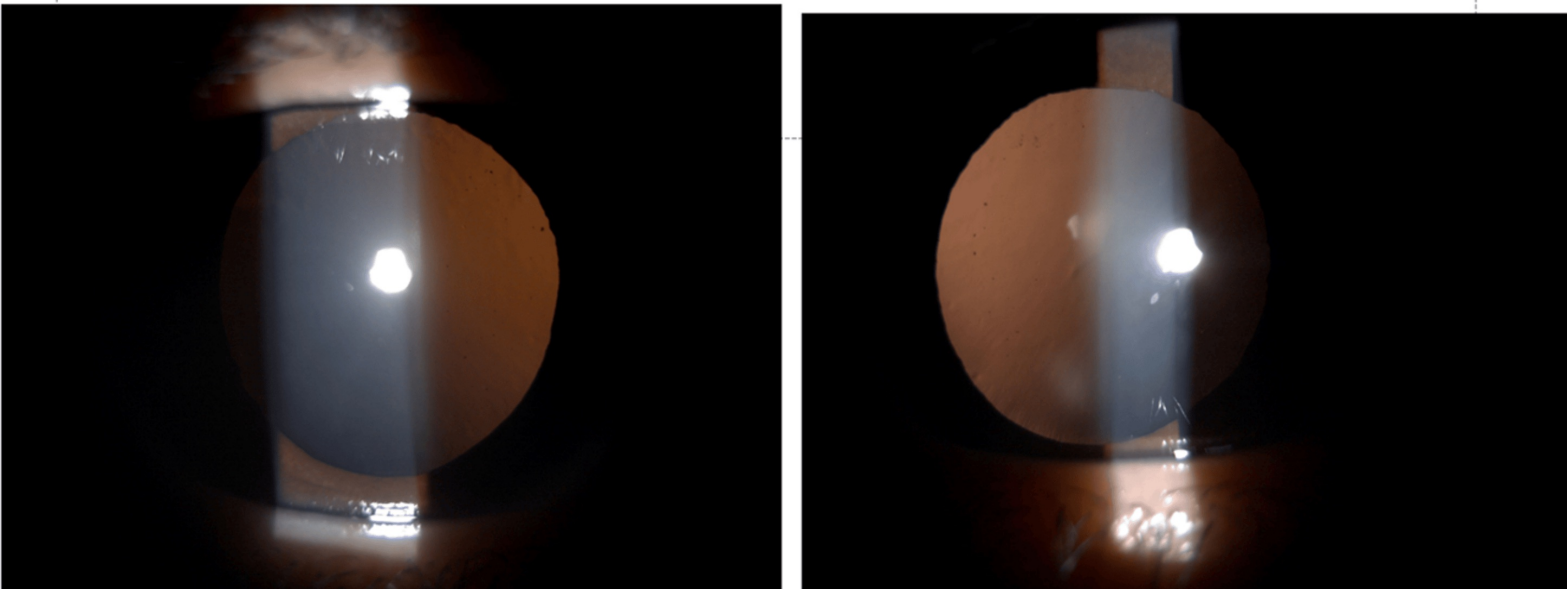
Close-up photographs with transillumination technique showing the absence of iris transillumination defects, along with the presence of pigmented iris clumps on the anterior capsule of the lens.

She presented to our service with an unremarkable medical and surgical history. There was no personal or family history of migraine. The patient denied any systemic complaints or focal neurological deficits and reported no prior history of head or ocular trauma. Additionally, she reported no use of new products or treatments that might affect her pupil size. She denied caffeine or alcohol intake and reported no use of illicit medications. The patient works in an office with no exposure to toxic materials and noted no changes in her diet or physical activities. Distance visual acuity was 0.1 logMAR (20/25) and near visual acuity was 0.3 logMAR (20/40) in both eyes. IOP measured 17 mmHg in the right eye and 18 mmHg in the left eye. Ocular motility was full, with intact saccades and pursuits, and no nystagmus was noted. Her lids were unremarkable and symmetrical, with no ptosis or lid retraction. The anterior and posterior segments appeared quiet, with no inflammation, although the anterior segment did have small clumps of pigment on the anterior lens surface. Pupil size was 6 mm bilaterally, with no reaction to light or near stimuli, and there was no vermiform movement of the iris or iris transillumination defects observed on slit lamp examination. Diluted pilocarpine (0.1%) and full-concentration pilocarpine (1%) did not affect pupil size (no tonic response). The patient declined iris fluorescein angiography because she had a prior fundus fluorescein angiography, which was complicated by nausea and vomiting. A contrast magnetic resonance imaging (MRI) of the brain and orbits was unremarkable. Laboratory investigations showed normal results for glycosylated hemoglobin, thyroid profile, syphilis serology, anti-ganglioside antibodies, nutritional panel, and toxic and heavy metal screening. The patient was assessed over a year of follow-up, during which her pupil size remained stable but dilated in both eyes. Diluted pilocarpine (0.1%) and full-concentration pilocarpine (1%) were again tried during follow-up visits, with no tonic response observed. The patient was prescribed hyperopic correction for near tasks, along with filtered lenses (FL-41), which significantly improved her blurred vision and photophobia. The patient preferred not to use colored contact lenses and declined any surgical intervention to reduce her pupil size (Figure 3).

## Discussion

A dilated pupil may be due to a wide range of pathologic processes. It can manifest secondary to isolated ocular dysfunction or abnormalities at several levels in the central or autonomic nervous system. Normal pupil size depends on the balance of the parasympathetic and sympathetic innervation to the iris muscles [5]. A dilated pupil indicates either sympathetic overstimulation or, more commonly, a dysfunction of the parasympathetic innervation of the pupil, which normally causes pupillary constriction. It is well known that the parasympathetic fibers for the pupil originate in the rostral midbrain at the level of the paired Edinger-Westphal nuclei, from where the fibers traverse the midbrain and emerge from the brainstem as part of the oculomotor nerve fascicle, extending through the nerve course. These pupillary fibers synapse in the ciliary ganglion, and the postsynaptic fibers then enter the globe to supply the iris sphincter muscle and the ciliary body [5]. Many supranuclear and nuclear abnormalities, including compression, inflammation, or traumatic injury, can result in a dilated atonic pupil as a part of parasympathetic pathway dysfunction, highlighting the need for urgent detailed imaging to rule out life-threatening conditions [6]. Migraine is an example that represents the imbalance between the autonomic nervous system, which can lead to either episodic benign pupillary mydriasis or recurrent painful ophthalmoplegic neuropathy, that is most likely secondary to parasympathetic hypoactivity and exhaustion [7]. Additionally, dilated pupils can occur due to exposure to pharmacologic or toxic agents that act on the central nervous system or peripheral autonomic system, including atropine, scopolamine, amphetamine, and botulinum toxins [5]. Hence, a thorough history and full neurological examination are necessary, in addition to laboratory investigations for toxic substances [5]. 

However, a dilated pupil can also occur from an injury at the level of the synapse at the ciliary ganglion or the postsynaptic fibers, which results in Adie’s tonic pupil. In this condition, the dilation of the pupil is not fixed but rather tonic, which can be observed by light-near dissociation and confirmed by low-dose cholinergic agents such as pilocarpine, leading to tonic pupillary constriction secondary to denervation hypersensitivity of the acetylcholine receptors on the sphincter muscle [8]. Orbital pathologies at the level of the ciliary ganglion have been associated with tonic pupils, including orbital tumors, orbital ischemia (including ischemia secondary to giant cell arteritis), and orbital trauma or postorbital surgical repair [9,10]. Additionally, injury to postsynaptic ciliary nerve fibers secondary to panretinal photocoagulation can lead to the same tonic pupillary response [11].

Besides the nerve supply to the pupillary sphincter, there are other important substructures, including the smooth sphincter muscle and its blood supply. A previous history of ocular trauma or complicated ocular surgery is a well-known risk factor for developing a dilated pupil due to sphincter muscle rupture and tear, warranting specific questions during history taking for these patients [12]. However, blood supply is crucial for preserving the function of the sphincter muscle. This observation has been confirmed by prior reports where iris angiography indicated pupillary dilation and iris atrophy secondary to defective blood supply to the iris sphincter muscles [13,14]. Anterior segment ischemia, which is commonly reported concurrently after strabismus surgery, can lead to a variety of signs that include fixed and dilated pupil, in addition to anterior segment inflammation and elevated IOP, which occur due to insufficiency of the anterior segment circulation caused by the manipulation of the recti muscles during strabismus surgery [15].

In 1963, Alberto Urrets-Zavalia reported six patients with dilated and fixed pupils after uneventful penetrating keratoplasty for keratoconus [1]. Over time, additional findings were reported in association with this abnormality, including iris atrophy, iris ectropion, and iris pigment dispersion [16]. The underlying mechanism that led to the dilated pupils was not clear at that time, and multiple theories were suggested [1]. Subsequently, numerous reports documented the same findings of Urrets-Zavalia syndrome following various ocular surgeries, including non-penetrating keratoplasty, cataract extraction, intraocular lens implants, gas injection into the anterior chamber, and trabeculectomy [17]. The common findings in the majority of those patients were that they were on mydriatic drops and experienced increased IOP postoperatively. Due to advances in ocular imaging, iris angiography showed segmental and incomplete filling, indicating remarkable iris ischemia in these cases, which explained this abnormal association [16,18,19]. The increased IOP is known to reduce vascular perfusion either to the anterior or posterior segment, including the optic nerve, explaining the non-responsive dilated pupil and the associated dispersed iris pigment clumps in the setting of acute angle-closure glaucoma with very high IOP [20]. 

To a lesser extent, dilated and fixed pupils have been reported in the setting of negative ocular interventions. An interesting report documented a patient with sickle cell thalassemia who presented with high IOP complicated by an abnormally dilated pupil [14]. Iris fluorescein angiography confirmed iris ischemia, which was attributed to the compound risk of sickle cell and elevated IOP, and the patient was classified as having Urrets-Zavalia-like syndrome [14]. In the setting of uveitis, only one case series in the literature reported persistent pupillary dilation in about 70% of patients with herpes simplex uveitis [2]. The authors presumed that the perineural invasion of the ciliary nerves by the virus particles, in addition to iris ischemia secondary to uveitic glaucoma, were the major risk factors for this association [2]. To the best of our understanding, there appears to be limited literature specifically addressing the association of dilated pupils with HLA-B27 uveitis. HLA-B27-associated uveitis manifests as an acute, anterior, and recurrent condition, typically demonstrating near-complete remission between attacks. Nonetheless, this etiology stands as the predominant cause of non-infectious uveitis on a global scale [4].

## Conclusions

To conclude, persistent pupillary dilation can be caused by numerous etiologies that can be simplified into neurologic or non-neurologic categories. Hence, neuro-ophthalmologists need to pursue a detailed history and examination, in addition to radiographic and ancillary laboratory investigations, in any patient with dilated and fixed pupils. It is essential to consider two factors: firstly, to differentiate whether the dilated pupil is tonic or atonic, and secondly, that the blood supply to the iris sphincter muscle is as important as the nerve supply, in which case, iris fluorescein angiography should be considered in the setting of a negative neurological work-up. We believe the administration of mydriatic drops, in conjunction with the combined effects of anterior segment inflammation and ocular hypertension, eventually led to iris ischemia, resulting in a dilated and atonic pupil. This case underscores the importance of careful IOP monitoring and judicious use of mydriatic agents in this patient population. The limitations of our study include the fact that this is a report of one case and the absence of iris angiography to confirm our suspected assumption of iris ischemia.
